# Feasibility and efficacy of therapeutic drug monitoring of abiraterone in metastatic castration resistant prostate cancer patients

**DOI:** 10.1038/s41416-025-02954-1

**Published:** 2025-02-11

**Authors:** Maud B. A. van der Kleij, Marinda Meertens, Stefanie L. Groenland, Sil Kordes, Andries M. Bergman, Jeantine M. de Feijter, Alwin D. R. Huitema, Neeltje Steeghs, on behalf of the Dutch Pharmacology Oncology Group (DPOG), Marinda Meertens, Stefanie L. Groenland, Alwin D. R. Huitema, Neeltje Steeghs, Maud B. A. van der Kleij

**Affiliations:** 1 Department of Clinical Pharmacology, Division of Medical Oncology, The Netherlands Cancer Institute, Antoni van Leeuwenhoek, Amsterdam, The Netherlands; 2 Department of Medical Oncology, Erasmus MC Cancer Institute, Rotterdam, The Netherlands; 3 Department of Pharmacy & Pharmacology, The Netherlands Cancer Institute, Antoni van Leeuwenhoek, Amsterdam, The Netherlands; 4 Department of Medical Oncology, The Netherlands Cancer Institute, Antoni van Leeuwenhoek, Amsterdam, The Netherlands; 5 Department of Clinical Pharmacy, Utrecht University Medical Centre, Utrecht University, Utrecht, The Netherlands; 6 Department of Pharmacology, Princess Máxima Centre for Paediatric Oncology, Utrecht, The Netherlands; 7 Department of Medical Oncology, Utrecht University Medical Centre, Utrecht University, Utrecht, The Netherlands

**Keywords:** Prostate cancer, Outcomes research, Targeted therapies

## Abstract

**Background:**

Previous studies demonstrated better outcomes for mCRPC (metastatic castration resistant prostate cancer) patients with higher abiraterone exposure (minimal plasma concentration (C_min_) > 8.4 ng/mL), but around 40% of patients experience exposure below this target. Pharmacokinetic (PK)-guided interventions following Therapeutic Drug Monitoring (TDM) could optimise exposure and outcomes. We aimed to evaluate the feasibility and effect on treatment outcomes of abiraterone TDM.

**Methods:**

Patients with low exposure levels (Low-group, C_min_ < 8.4 ng/mL) got a PK-guided intervention. We compared exposure, adverse event (AE) incidence, time on treatment (ToT) and Prostate-Specific Antigen response rate (PSArr) between the Low-group and Adequate-group.

**Results:**

We included 167 mCRPC patients, with 56 in the Adequate-group and 111 in the Low-group. Interventions were successful 86% of the time. Exposure between groups became corresponding (Low-group: 7.95 to 20.5 ng/mL, Adequate-group: 20.8 ng/mL, *p* = 0.72) with comparable AE incidence (17% vs. 23%, *p* = 0.4). Median ToT and PSArr were similar (351 vs. 379 days, *p* = 0.35; 61.3% vs. 67.9%, *p* = 0.51).

**Conclusions:**

PK-guided interventions improved above target exposure from 33.5% to 81.4% of patients without additional AEs. While historically, low exposure patients had significantly shorter survival, PK-guided interventions eliminated this disparity. As interventions are effective, low-cost and safe, TDM for abiraterone should be considered to enhance treatment outcomes.

## Background

Prostate cancer is the second most frequent form of cancer in males. Over a million men are diagnosed each year [1]. As prostate cancer growth depends on testosterone, the cornerstone of treatment in metastatic disease is androgen deprivation therapy (ADT). ADT inhibits the production of testicular androgens and results into circulating testosterone at the castration level ( < 50 ng/dL). Virtually, all patients initially respond to ADT and the disease is labelled as metastatic hormone sensitive prostate cancer (mHSPC). However, resistance inevitably develops as metastatic castration resistant prostate cancer (mCRPC) [2]. There are multiple treatment options for mCRPC patients, including chemotherapy, radionuclides and androgen receptor signalling inhibitors (ARSIs).

Abiraterone acetate is an ARSI, which irreversibly inhibits cytochrome P450 17α (CYP17). This is an important enzyme in androgen synthesis not only expressed in the testes, but also in prostatic tumour tissue and the adrenal glands [3, 4]. Abiraterone acetate in combination with ADT results in undetectable low serum testosterone levels and is combined with a corticosteroid to inhibit a rise in ACTH resulting in a mineralocorticoid excess and possible forthcoming adverse events (AEs) (combined treatment from now on: abiraterone) [5]. Abiraterone was approved as first-line treatment and as second-line treatment after docetaxel-based chemotherapy for patients with mCRPC in the early 2010’s and for patients with high-risk mHSPC in 2018 [5, 6]. Use of abiraterone increased progression free survival (PFS) in mCRPC patients from 8.3 months to 16.5 months in first-line treatment [7] and in patients previously treated with chemotherapy PFS increased from 3.6 months to 5.6 months [8].

Previous studies have shown that abiraterone exposure is related to efficacy. In two studies including both chemotherapy naïve and chemotherapy pre-treated patients, minimal plasma concentrations at steady-state (C_min_) of ≥8.4 ng/mL led to longer PFS: 12.2 vs. 7.4 months, *p* = 0.044 (*n* = 55) and 16.9 vs. 6.1 months, *p* = 0.077 (*n* = 62) [9, 10]. Although abiraterone shows high interindividual variability in exposure (coefficient of variation of 45–70%), and many patients have exposure below the target of 8.4 ng/mL (35–42%) [9, 10], patients are still treated with a standard dose of 1000 mg once daily (OD) fasted. Therapeutic drug monitoring (TDM) - treatment and dose adjustments based on pharmacokinetic (PK) levels (PK-guided interventions) and set targets - could improve exposure levels and potentially also improve individual treatment outcomes. Because abiraterone food-effect studies showed that intake with food results in a clinically relevant increase in exposure, food interventions could be a possible effective PK-guided intervention [11, 12].

In this study, we aimed to evaluate the effect of TDM of abiraterone targeting a C_min_ ≥ 8.4 ng/mL on treatment outcomes for mCRPC patients, by comparing patients with at least one low exposure level who, thus, were eligible for an intervention, to patients who had all adequate exposure levels. For this purpose, we examined differences in Prostate Specific Antigen response rate (PSArr), time on treatment (ToT) and incidence of adverse events (AEs). We also assessed the practical feasibility of TDM.

## Methods

### Study design and patient selection

Patients from the Netherlands Cancer Institute (NKI) who started treatment with abiraterone 1000 mg OD in a modified fasted state (according to drug label) between June 2017 and December 2021 were retrospectively selected for this analysis. They were included from an ongoing multicentre prospective trial on TDM of oral targeted therapies used in oncology (Dutch Pharmacology Oncology Group – Therapeutic Drug Monitoring study (DPOG – TDM study), https://trialsearch.who.int/, trial number NTR6866) [13]. Only patients with mCRPC were eligible for this analysis, so mHSPC patients were excluded. In- and exclusion criteria are further elucidated in the published protocol [13]. The DPOG-TDM study was approved by the Institutional Research Board of the NKI (reference number: IRBd20-011). All patients provided written informed consent. Data on baseline characteristics, dosing, PK levels, recommended and performed PK-guided interventions and reasons if not performed or not successful, tumour evaluation, end of treatment, follow-up and clinical relevant treatment related AEs (AEs leading to treatment discontinuation, dose reductions, treatment interruptions or restricting PK-guided interventions) (trAEs) (according to the Common Terminology Criteria for Adverse Events grading system version 5) were collected. We considered patients evaluable when ≥1 PK level was measured, and intervention was considered evaluable when there was ≥1 PK level before- and ≥1 PK level after intervention. Data cut-off was the 11^th^ of June 2023.

### PK levels and PK-guided interventions

PK levels were drawn 4, 8, and 12 weeks after treatment start, and every 12 weeks thereafter (following clinical practice at the NKI). PK levels were measured using a validated liquid chromatography-tandem mass spectrometry assay [14]. Date and time of last abiraterone intake were recorded. PK levels were preferably taken predose to retrieve a C_min_. When a C_min_ measurement was not possible, C_min_ was estimated using log-linear extrapolation [15, 16]. In February 2020 this method was switched to calculating the ratio of the measured PK level and the typical population concentration, and multiplying this ratio with the typical population C_min_ value, as it was proven to give better estimations [15]. PK-guided interventions were recommended based on a set PK-target C_min_ at steady-state of 8.4 ng/mL [9]. Interventions were only carried out if deemed feasible by the treating physician considering toxicity. Recommended PK-guided interventions when a PK level was <8.4 ng/mL included switching from a fasted state to taking the abiraterone with the smallest meal of the day and if not successful, dose increments from 1000 mg OD to 1500 mg OD. A successful intervention was defined as median exposure after intervention above target and no dose-limiting toxicity within one month following intervention.

### TDM groups

Patients were divided into TDM groups based on their PK levels following intention-to-treat analysis. Patients were considered Adequate-group if they had all PK levels ≥8.4 ng/mL. Patients were considered Low-group if they had ≥1 PK level <8.4 ng/mL, including patients with and without an intervention, and independent of successfulness of the intervention. To evaluate the influence of the intervention on exposure, PK levels were evaluated within the subgroup that got an intervention and were evaluable ( ≥ 1 measured PK level after intervention), differentiating between exposure before and after intervention.

### Aim of the study

The primary aim of the study was to evaluate the effect of PK-guided interventions following TDM on efficacy, by comparing the group with low abiraterone exposure levels and, thus, eligible for a PK-guided intervention, to the group with adequate abiraterone exposure levels and, thus, not eligible for a PK-guided intervention, with ToT and PSArr as efficacy surrogates. ToT was defined as day from start of abiraterone, until the last day of therapy. ToT as surrogate for efficacy was expected to give the best representation of clinical practice and the physicians’ decision to continue or to discontinue treatment because of progression based on clinical or radiological progression or PSA increase. These are a reflection of factors described in the Prostate Cancer Working Group 3 consensus for trials in CRPC used in the real world [17]. Patients who stopped therapy due to other causes or who still used abiraterone at the last day of follow-up were censored at either the time of discontinuation or the last day of follow-up. PSArr was defined as ≥50% decrease in PSA from baseline, following the Prostate Cancer Working Group 3 criteria [17]. In previous studies, patients with drug exposure levels <8.4 ng/mL had shorter PFS [9, 10], and we hypothesised that our PK-guided interventions targeting exposure levels >8.4 ng/mL would result in now similar efficacy levels for patients with low exposure levels (who were eligible for an intervention) and patients with all adequate exposure levels.

Secondary aims were to halve the proportion of patients with a low PK level at the third measurement compared to historical data, and to evaluate feasibility and toxicity of TDM. The reference from historical data for the proportion of patients with a low PK level was 38.5%, set as a combined result from the study of Carton et al. in which 35% of patients (*n* = 61) had exposure <8.4 ng/mL, and the study of van Nuland et al, where 42% of patients (*n* = 62) had exposure <8.4 ng/mL [9, 10]. Feasibility was defined as the percentage of successful PK-guided interventions. Results on the first 30 patients regarding feasibility and preliminary efficacy results were previously published elsewhere [11], just as results on feasibility of TDM for 79 abiraterone patients as part of a larger analysis [18]. Tolerability was defined as clinically relevant treatment related AEs (following the Common Terminology Criteria for Adverse Events (CTCAE) grading system, version 5.0) following TDM, and was compared between TDM groups. Clinically relevant adverse events include adverse events that led to interruption of abiraterone treatment, discontinuation of abiraterone treatment, dose reductions or limiting food- or dose interventions.

### Statistical analyses

Baseline characteristics were compared between TDM groups using a Mann-Whitney U test or a Fisher exact test. Exposure in a TDM group was defined as the median of the median exposure (seen as C_min_) calculated per patient, and differences between groups were evaluated with a Wilcoxon-signed rank test. ToT was evaluated using Kaplan-Meier survival analysis, and compared with a log-rank test. PSArr was compared with a two-proportion z-test. Univariate and multivariate Cox regression analysis (ToT and logistic regression analysis (PSArr)) were executed for the TDM groups and included acknowledged factors influencing abiraterone efficacy: age, WHO (World Health Organization) performance status, Gleason score, PSA, metastases location, number of previous treatment lines in mCRPC phase, previous chemotherapy in CRPC phase and previous chemotherapy in HSPC phase. A Schoenfeld Test was performed to test the Cox model. Hazard ratios are stated with the 95% confidence interval. Incidence of clinically relevant TrAEs was compared between TDM groups with a Fisher exact test. For all analyses, a *p*-value < 0.05 was considered significant. Other data was analysed using descriptive statistics. Statistical analyses were performed with R version 4.2.2 (R Project, Vienna, Austria).

## Results

### Patients

A total of 189 patients using abiraterone were included in the DPOG-TDM study, of which 22 patients were excluded for this analysis: thirteen patients were excluded because their PK levels were not evaluable because of stability issues, eight patients had mHSPC and one patient was excluded because he did not start on the dose according to label (see Supplementary Fig. 1). This resulted in 167 evaluable patients. The Adequate-group consisted of 56 patients (33.5%) and the Low-group consisted of 111 patients (66.5%). Baseline characteristics are described in Table 1. All baseline characteristics were statistically comparable between TDM groups. Of all 167 patients, 133 patients (79.6%) were off treatment at the time of analysis, because of progressive disease (*n* = 109; 82%), toxicity (*n* = 8; 6%), death of other cause (*n* = 6; 4.5%), or other reasons (*n* = 10; 7.5%).

**Table 1:** Baseline characteristics.

	All patients	Adequate	Low
Total patients	167	56	111
**Age at baseline (years)**	73 [67–76]	74 [69.8–77]	73 [66.5-76]
**WHO performance status at baseline**			
WHO 0	47 (28.1)	18 (32.1)	29 (26.1)
WHO 1	72 (43.1)	21 (37.5)	51(46.0)
WHO 2	22 (13.2)	8 (14.3)	14 (12.6)
WHO 3	2 (1.2)	1 (1.8)	1 (0.9)
Unknown	24 (14.4)	8 (14.3)	16 (14.4)
**Gleason score at initial diagnosis**			
5	2 (1.2)	2 (3.6)	0 (0)
6	14 (8.4)	4 (7.1)	10 (9.0)
7	55 (32.9)	18 (28.6)	37 (33.3)
8	39 (23.4)	16 (28.6)	23 (20.7)
9	46 (27.5)	14 (25.0)	32 (28.8)
10	6 (3.6)	1 (1.8)	5 (4.5)
Unknown	5 (3.0)	1 (1.8)	4 (3.6)
**PSA (ng/mL) at baseline**	26.9 [8.7–92.5]	28.7 [7.7–66.2]	25.4 [9.5–96.7]
**Location of metastases at baseline**			
Bone	143 (85.6)	51 (91.1)	92 (82.9)
Node	95 (56.9)	29 (51.8)	66 (59.5)
Visceral	22 (13.2)	8 (14.3)	14 (12.6)
**Switch from prednisone to dexamethasone during study (yes)**	97 (58.1)	30 (53.6)	67 (60.4)
**Number of previous lines of therapy in CRPC phase**			
0	129 (77.2)	45 (80.4)	84 (75.7)
1	12 (7.2)	3 (5.35)	9 (8.1)
2	11 (6.6)	3 (5.35)	8 (7.2)
3	5 (3.0)	2 (3.6)	3 (2.7)
≥4	10 (6)	3 (5.4)	7 (6.3)
**Previous lines of therapy in CRPC phase**			
enzalutamide	23 (10.8)	8 (14.3)	15 (13.5)
docetaxel	22 (13.2)	5 (8.9)	17 (15.3)
cabazitaxel	18 (10.8)	5 (8.9)	13 (11.7)
radium-223	9 (5.4)	3 (5.4)	6 (5.4)
abiraterone	3 (1.8)	1 (1.8)	2 (1.8)
other chemotherapy	18 (10.8)	5 (8.9)	13 (11.7)
other therapy	10 (6)	3 (5.4)	7 (6.3)
**Received chemotherapy during CRPC phase (yes)**	28 (16.8)	7 (12.5)	21 (18.9)
**Received chemotherapy during HSPC phase (yes)**	30 (18)	8 (14.3)	22 (19.8)

Data are expressed as number (percentage) or median [interquartile range]. Due to rounding, total percentages could be deviating from 100%. Adequate: patients with all pharmacokinetic levels ≥8.4 ng/mL. Low: patients with ≥1 pharmacokinetic level <8.4 ng/mL.

*WHO* World Health Organization, *PSA* Prostate-Specific Antigen, *ng/mL* nanogram per millilitre, *CRPC* castration resistant prostate cancer, *HSPC* hormone sensitive prostate cancer

### PK exposure

**Fig. 1:**
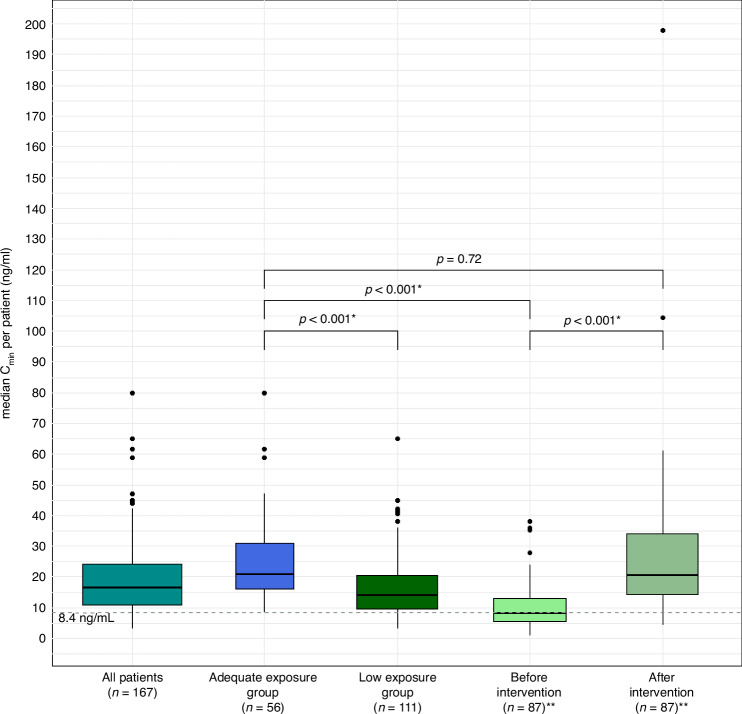
Median exposure per group (C_min_). Boxplots of median C_min_ per patient in all patients, the Adequate- and the Low-group, also shown divided in a before and after intervention subgroup. Adequate exposure group: patients with all pharmacokinetic levels **≥**8.4 ng/mL. Low exposure group: patients with ≥1 pharmacokinetic level <8.4 ng/mL. Before intervention: patients in the Low-group before intervention. After intervention: patients in the Low-group after intervention. *: significant (*p*-value < 0.05). **: only including patients who had an evaluable intervention. *C*_min_ minimal plasma concentrations at steady-state.

We measured a total of 1234 PK levels from 167 patients, with a median of 7 samples per patient (IQR 4-11). Median exposure (median C_min_) while taking the starting dose of 1000 mg OD fasted was 12.0 ng/mL (IQR: 7.2-20.7 ng/mL) (see supplementary Fig. 2 for the exposure per patient at the starting dose). Median abiraterone plasma concentration off all samples of all patients was 16.5 ng/mL. The median exposure in the Low-group in patients who got an evaluable intervention was significantly higher after the intervention than before the intervention (20.5 ng/mL vs. 7.95 ng/mL, *p* < 0.001). Exposure in the Adequate-group was significantly higher than exposure in the Low-group before intervention (20.8 ng/mL vs. 7.95 ng/mL, *p* < 0.001), but similar as exposure in the Low-group after evaluable intervention (20.8 ng/mL vs. 20.5 ng/mL, *p* = 0.72). Figure 1 and Table 2 provide an (visual) overview of the exposure in- and between groups.

**Table 2:** Median exposure per group (C_min_).

	All patients	Adequate	Low
**Total patients**	167	56	111
**PK samples total**	1234	392	842
**PK samples per patient**	7 [4–11]	7 [3.8-9]	6 [4–11]
**Median exposure (ng/mL)**	16.5 [11.0–24.1]	20.8 [16.0–30.9]	14 [9.5–20.4]
**Median exposure before intervention (ng/mL)**	NA	NA	7.95 [5.65–13.0]*
**Median exposure after intervention (ng/mL)**	NA	NA	20.5 [14.3–34.0]*

Data are expressed as number or as median [interquartile range]. *: only including patients who had an evaluable intervention (*n* = 86). Adequate: patients with all pharmacokinetic levels ≥8.4 ng/mL. Low: patients with ≥1 pharmacokinetic level <8.4 ng/mL.

*C*_*min*_ minimum plasma concentration at steady-state, *PK* pharmacokinetic, *ng/mL* nanogram per millilitre.

### Feasibility of PK-guided interventions

**Fig. 2:**
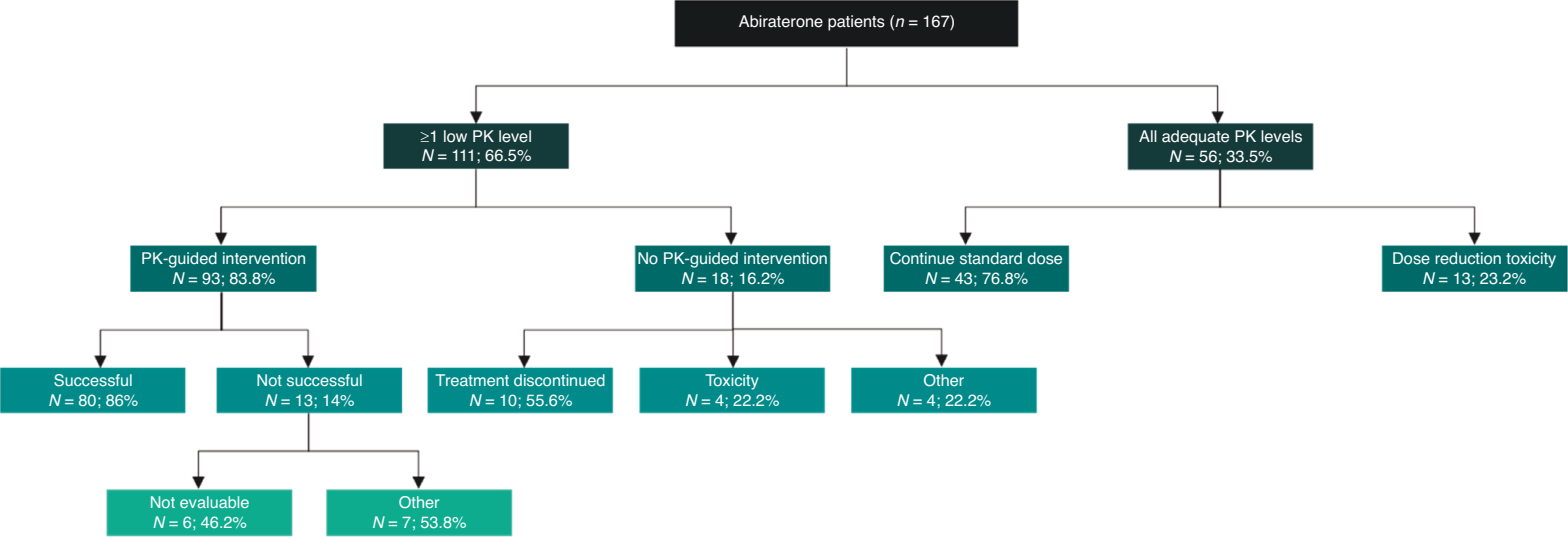
Schematic overview of the feasibility of PK-guided interventions. Data are expressed as number (N) and percentage (%). Patients had ≥1 low PK level or all adequate PK levels. Patients with ≥1 low PK level did or did not have a PK-guided intervention. This PK-guided intervention was either successful or not successful. If patients had no PK-guided intervention, the reason why was stated. If patients had all adequate PK levels, it was stated if the standard dose was continued throughout the study, or dose reduction/dose interruption/end of treatment was necessary as a result of treatment related toxicity. PK pharmacokinetic.

Of the 111 patients in the Low-group, 93 patients got a PK-guided intervention (83.8%). Out of the 111 patients in the Low-group, for 83 patients the first low exposure level was measured during one of the first three PK levels. Out of the 93 patients who received an intervention, this was the case in 72 patients. Most often the intervention was a food intervention (*n* = 92); 13 patients had an (additional) dose increase. For 12 patients this was an increase to 1500 mg OD with food, but for one patients this was an increase from 500 mg OD to 1000 mg OD fasted after previous dose decrease following toxicity. The PK-guided intervention was successful in 80 out of 93 patients (86%), and not successful in 13 patients (14%), in almost half (*n* = 6) this was because there was no PK level after the intervention so the intervention was not evaluable (*n* = 6, 46.2%). If we excluded the six patients for whom the intervention was not evaluable, the intervention was successful in 80 out of 87 patients, so 93% of patients. From the 18 patients (16.2%) who did not get a PK-guided intervention, for 10 patients this was due to treatment discontinuation after the low PK level. Overall, including all patients in the Low-group whom were evaluable, 76.1% had a successful intervention. PK-guided interventions increased the proportion of patients with PK-levels above target with 47.9% (33.5% to 81.4%). For a full overview of the feasibility of PK-guided interventions, see Fig. 2. We met our secondary aim to halve the proportion of patients with a low PK level at the third measurement compared to historical data (18.5% vs. 38.5%, *p* < 0.001).

### Efficacy of TDM

**Fig. 3:**
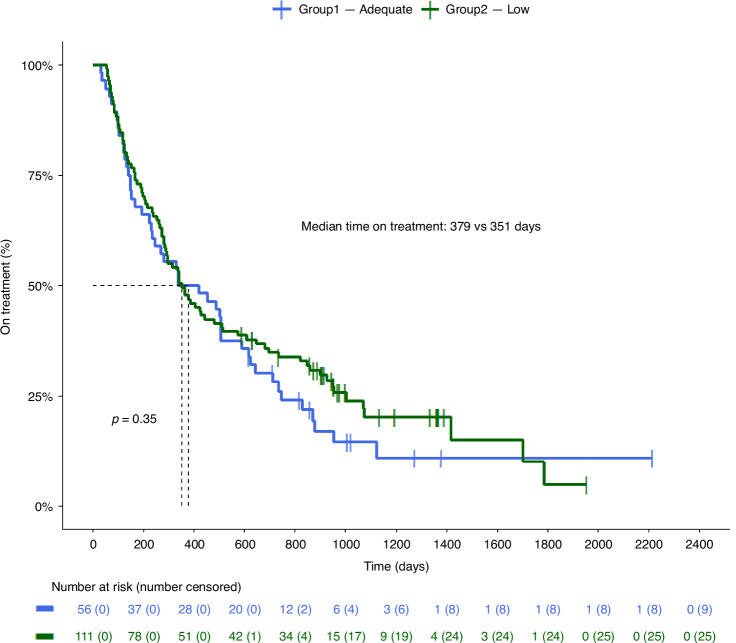
Kaplan-Meier curves illustrating Time on Treatment in the Adequate-group vs. the Low-group. Kaplan-Meier curves of the Adequate- and the Low-group. Adequate: patients with all pharmacokinetic levels **≥**8.4 ng/mL. Low: patients with ≥1 pharmacokinetic level <8.4 ng/mL. *P*-value was calculated with log-rank.

Median ToT for all patients was 351 days (95% CI: 286–504 days). For the Adequate-group this was 379 days (95% CI: 234–615 days) and for the Low-group 351 days (95% CI: 286–514 days), which was not significantly different (*p* = 0.35) (Fig. 3). In the univariate Cox regression analysis, WHO performance status at baseline, baseline PSA, number of previous lines of therapy in CRPC phase and previous chemotherapy in the CRPC phase appeared to be prognostic factors; in the multivariate analysis there were no prognostic values (Table 3). Comparing TDM groups, HR in the univariate analysis was 0.84 (CI 95% 0.5–1.12, *p* = 0.4) and HR in the multivariate analysis was 0.79 (CI 95% 0.52–1.2, *p* = 0.3), in favour of the Low-group. PSArr was similar between the Adequate-group and the Low-group (67.9% vs. 61.3%, *p* = 0.51). Multivariate logistic regression analysis showed that the Low-group had an odds ratio for PSA response of 0.91 compared to the Adequate-group (*p* = 0.25).

**Table 3:** Univariate and multivariate cox-regression analysis on Time on Treatment.

Variable	Univariate analysis			Multivariate analysis		
	HR	95% CI	*P* value	HR	95% CI	*P* value
**Low- vs. Adequate-group**	0.84	0.59–1.2	0.4	0.79	0.52–1.2	0.3
**Age at baseline: >median vs. ≤median**	0.75	0.52–1.1	0.1	1.1	0.68–1.8	0.9
**WHO performance status at baseline: 2-3 vs. 0-1**	1.9	1.2–3.0	0.007*	1.3	0.78–2.3	0.2
**Gleason score at initial diagnosis: >7 vs. ≤7**	1.3	0.92–1.9	0.1	1.4	0.89–2.2	0.09
**Baseline PSA: >median vs. ≤median**	1.5	1.1–2.2	0.01*	1.4	0.91–2.3	0.2
**Location of metastases at baseline:**						
Bone vs. no-bone	1.7	0.99–3.0	0.05	1.6	0.85–3.1	0.1
Node vs. no-node	1.2	0.8–1.7	0.4	1.1	0.69–1.7	0.9
Visceral vs. no-visceral	1.3	0.8–2.1	0.3	1.7	0.97–3.2	0.1
**Number of previous lines of therapy in CRPC phase: >0 vs. 0**	3.3	2.2–5.0	<0.001*	2.3	0.95–5.6	0.05
**Received chemotherapy during CRPC phase: yes vs. no**	4.0	2.6–6.4	<0.001*	2.6	0.97–7.0	0.1
**Received chemotherapy during HSPC phase : yes vs. no**	1.4	0.89–2.1	0.2	1.8	1.0–3.2	0.1

*: significant (*p*-value<0.05). Adequate: patients with all pharmacokinetic levels ≥8.4 ng/mL. Low: patients with ≥1 pharmacokinetic level <8.4 ng/mL.

*HR* Hazard Ratio, *95% CI* 95% confidence interval, *WHO* World Health Organization, *PSA* Prostate-Specific Antigen, *CRPC* castration resistant prostate cancer, *HSPC* hormone sensitive prostate cancer.

### Toxicity of TDM

Considering all patients, 32 patients experienced clinically relevant trAEs. There was no significant difference in clinically relevant trAE incidence between the Adequate- and Low-group (*n* = 13 (23%) vs. *n* = 19 (17%), *p* = 0.4). The most common clinically relevant trAE was increased alanine aminotransferase levels (ALT) (*n* = 7). For seven out of 19 patients in the Low-group, interventions following a low PK level were limited because patients already experienced trAEs. For six out of the 19 patients, trAEs occurred within three months after the intervention and lead to treatment interruption (*n* = 2) or end of treatment (*n* = 1 without progression, *n* = 3 for patients also having progressive disease). Patients experienced fatigue grade 2 (*n* = 2), increase of ALT grade 2 (*n* = 2) and grade 3 (*n* = 1) and chest pain grade 2 (*n* = 1). One out of the 19 patients experience dyspnoea grade 2 a year after intervention, which let to treatment interruption. For the last five patients, trAEs occurred on the standard dose. For them, after the trAEs resolved, an intervention was possible. This was successful for four patients and not evaluable for one patient.

## Discussion

In this study, we investigated TDM of abiraterone in mCRPC patients, and elucidated the feasibility of PK-guided interventions and their influence on time on treatment, PSA response rate and adverse events incidence. Around 66% of patients experienced low abiraterone exposure, of which 84% had a PK-guided intervention. PK-guided interventions increased the proportion of patients with adequate PK-levels by 47.9% (from 33.5% to 81.4%), and resulted in a significant exposure increase. PK-guided interventions did not lead to more adverse events and PSArr and ToT were similar between groups.

PK-guided interventions were feasible to execute and successful. Food interventions were carried out in many patients in the Low-group (92 out of 111 patients), and only 13 patients needed a dose increase for a successful intervention. Overall, TDM led to adequate exposure in 76.1% of patients initially in need of an intervention (all patients in the Low-group), without additional AEs. Our definition of ≥1 low PK level to qualify for a PK-guided intervention (and to fall into the Low-group) was relatively strict, but pragmatic, as it was easy to follow and well-tolerated in clinical practice.

In previous research, low exposure levels of abiraterone were present in around 35–42% of patients [9, 10]. In our study, low exposure levels occurred in a much larger part of patients (66.5% of patients). This difference is, however, a difference in the definition of low exposure. In our study patients were divided into the Low-group when they had at least one PK level below target, and in these other studies patients were classified as underexposed when mean or median abiraterone levels were below target. In our study, only 12% of patients had a median C_min_ below target because of the successfulness of the interventions.

We found that ToT (*p* = 0.35) and PSArr (*p* = 0.25) between TDM groups were similar; ToT in the Adequate-group was 379 days (12.5 months), PSArr was 61.3%; ToT in the Low-group 351 days (11.5 months) and PSArr was 61.3%. Kaplan-Meier curves of these groups overlapped. In previous studies an exposure-response relationship was established, and Kaplan-Meier curves for PFS vs. months since start of treatment between patients with adequate and low exposure ( ≥ 8.4 ng/mL vs. <8.4 ng/mL) were significantly different (PFS 12.2 vs. 7.4 months, *p* = 0.077 and PFS 16.9 vs. 6.1 months, *p* = 0.044) [9, 10]. Exposure ≥8.4 ng/mL was associated with PSA response (*p* = 0.004) [9]. The vast majority of patients in our Low-group (84%) had a PK-guided intervention and exposure significantly increased. Exposure between TDM groups after intervention was comparable. Thus, this suggests that our interventions are responsible for the correction of the Kaplan-Meier curves and for the similarity in PSArr between groups. Because prior chemotherapy accords for worse efficacy outcomes, and more patients in the Low-group have had prior chemotherapy, this may limit efficacy comparisons. However, the differences in baseline characteristics were not statistical different, and in multivariate analysis chemotherapy pre-treatment was not a significant variable, so this small difference in baseline pre-chemotherapy treatment is most probably negligible.

Although one previous study found no exposure-response relationship for chemotherapy naïve patients – as many of the patients in our study are – this was not evident in another exposure-response relationship study [10, 19]. In addition, we should realise when interpreting these results that the risk of being classified as Low-group might increase with time, as the chance of a low PK level could increase with more measured PK levels due to the relatively high intra-individual variability (74.7%) [18]. Remarkably, this risk was not reflected in our data, as 83 out of 111 patients in the Low-group had a low PK level within the first three PK levels.

On the one hand, following evidence based medicine, a randomised controlled trial (RCT) is needed to confirm that PK-guided interventions can improve treatment outcomes. As we did not have a comparing non-intervention patient group, we cannot discern the benefits of our intervention. On the other hand, the question is if the relatively simple and cost-effective way of raising exposure with a food intervention could not be implemented without the need for an RCT. Up to now, no exposure-toxicity relationship for abiraterone has been established. We also did not observe additional toxicity with (food) interventions in our study; nor was it seen in a small study including 19 patients who received standard dose abiraterone with food [20]. As a result, the risk of this pragmatic approach is minimal, especially in an environment where PK levels are measured. Additionally, taking abiraterone with food is more convenient for patients, as there is no need for fasting [21].

Future research could focus on the necessity for the current 1000 mg OD fasted dose. In a small bio-equivalence PK cross-over study including 12 patients, PK between 500 mg OD fed vs. 1000 mg OD fasted was not bioequivalent in the 90% confidence interval following FDA guidelines, although it fell within the set thresholds [21]. In contrast, another study showed non-inferiority for 250 mg OD fed vs. 1000 mg OD fasted for PSA response after 12 weeks [22, 23]. However, this study did find pharmacokinetic inferiority between the groups, although neither groups had exposure ≥8.4 ng/mL [22–24]. Additional studies could further elucidate the effect of a lower abiraterone dose combined with food on exposure and efficacy. Although this research might be less relevant for some countries as abiraterone has gone out of patent [25], it could still be useful in countries with high medical costs or low abiraterone availability.

This study is the first to evaluate TDM of abiraterone in means of feasibility, influence on exposure, adverse events and efficacy. A limitation of the study is that we have no results on time to PSA progression, PFS and overall survival. Furthermore, the lack of a comparing non-TDM cohort limits the positive evidence of the influence of TDM on ToT and PSArr. A strength of this study is the good representation of clinical practice, following clinical decisions in the real world, with inclusion of all mCRPC patients treated with abiraterone.

## Conclusions

PK-guided interventions following TDM in mCRPC patients using abiraterone were feasible to execute and did not lead to additional adverse events. These interventions resulted in adequate PK levels of abiraterone in the majority of patients with initially low PK levels. While historically, patients with low exposure had significantly lower survival rates, we found that patients with initially low exposure who received successful interventions had comparable time on treatment and PSA response rates as those with adequate exposure levels, suggesting that our interventions played a crucial role in bridging this gap. As these interventions are effective, safe, simple and low cost, TDM should be implemented in routine clinical practice to enhance abiraterone exposure en therapy treatment outcomes for mCRPC patients.

## Supplementary information

Supplementary figure 1

Supplementary figure 2

## Data Availability

The data that support the findings of this study are available from the corresponding author upon reasonable request.
