# Supplementary material for: Feasibility and efficacy of therapeutic drug monitoring of abiraterone in metastatic castration resistant prostate cancer patients
Source: Br J Cancer. 2025 Feb 11;132(7):635–42. doi: 10.1038/s41416-025-02954-1 (PMC11961573; doi:10.1038/s41416-025-02954-1)
Supplement: Supplementary file 1 — Supplementary figure 1 [file 41416_2025_2954_MOESM1_ESM.docx]

**Supplementary Figure 1: Patient selection**

**
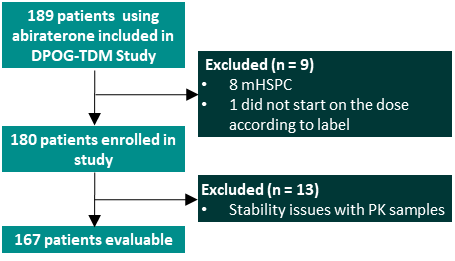
**

**Supplementary Figure 1**: patient selection. *DPOG-TDM: Dutch Pharmacology Oncology Group – Therapeutic Drug Monitoring Study; mHSPC: metastatic Hormone Sensitive Prostate Cancer; PK: pharmacokinetic.*
