# Supplementary material for: Feasibility and efficacy of therapeutic drug monitoring of abiraterone in metastatic castration resistant prostate cancer patients
Source: Br J Cancer. 2025 Feb 11;132(7):635–42. doi: 10.1038/s41416-025-02954-1 (PMC11961573; doi:10.1038/s41416-025-02954-1)
Supplement: Supplementary file 2 — Supplementary figure 2 [file 41416_2025_2954_MOESM2_ESM.docx]

**Supplementary Figure 2: Median exposure (C_min_) at starting dose of 1000mg OD fasted**


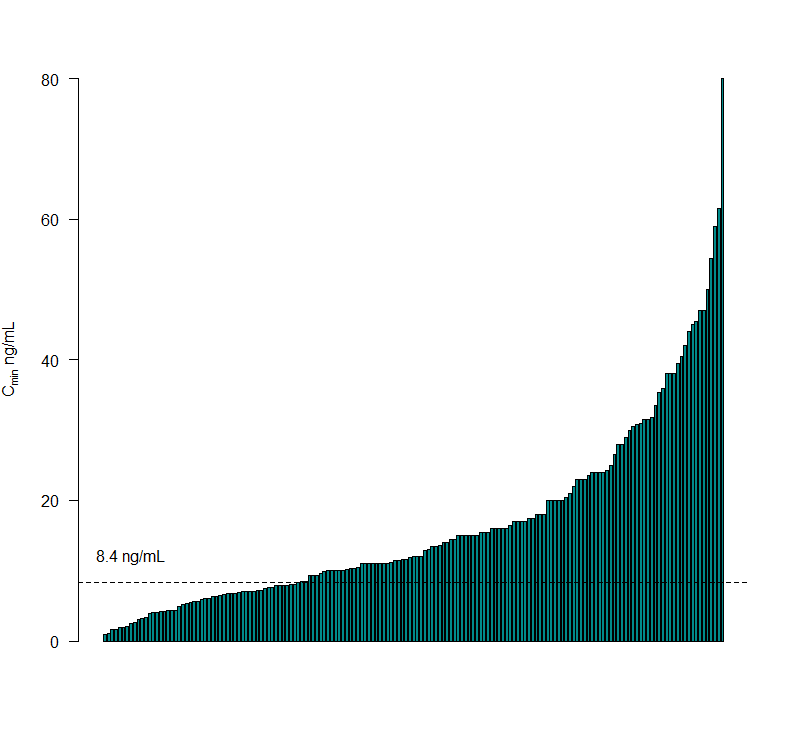


**Supplementary Figure 2**: Median C_min_ per patient at the starting dose of 1000mg OD fasted. Each bar represents one patient. The dashed line is the efficacy target of 8.4 ng/mL. *C_min_: minimal plasma concentrations at steady-state; ng/mL: nanogram per millilitre; OD: Once Daily*
